# Recent advances in artificial intelligence for radiology report generation: a brief review

**DOI:** 10.1093/bjrai/ubag003

**Published:** 2026-01-30

**Authors:** Caitlyn Tran, Basudha Pal, Thomas Stirrat, Victoria Shi, Muhammad Umair

**Affiliations:** Georgetown University School of Medicine, Washington, DC 20007, United States; Department of Electrical and Computer Engineering, Johns Hopkins University, Baltimore, MD 21218, United States; Georgetown University School of Medicine, Washington, DC 20007, United States; Russell H. Morgan Department of Radiology and Radiological Sciences, The Johns Hopkins Hospital, Baltimore, MD 21287, United States; Department of Radiology, Columbia University Irving Medical Center, New York, NY 10032, United States

**Keywords:** artificial intelligence, radiology report generation, vision-language models, natural language processing, deep learning, medical imaging, clinical AI, radiology automation, diagnostic accuracy, radiology workflows

## Abstract

Recent advances in artificial intelligence (AI) offer significant potential to address the growing bottleneck in radiology caused by an increasing volume of imaging studies amidst a global shortage of radiology professionals. This study presents a comprehensive review of the latest developments in AI, particularly in vision-language models for radiology report generation, providing radiologists with a current reference. We conducted a focused literature search for studies published from 2020 to 2024 and included 14 studies in our review specifically on chest X-ray datasets with limited coverage of 3D modalities, reflecting the early stage of research and ongoing methodological advances in report generation for volumetric imaging. We analysed the model architectures, report generation capabilities, training datasets, evaluation metrics, and performance of these models. Our review highlights the evolution of AI in radiology report generation and underscores the critical need for diverse datasets and standardized evaluation metrics. Despite rapid progress, current AI models are not yet capable of consistently producing high-quality reports and require further improvements in data diversity, model training, and evaluation metrics to achieve a level comparable to human experts.

Essentials

- Recent rapid developments necessitate this comprehensive review to help radiologists stay updated.
- Current AI models for generating radiology reports primarily utilize advanced vision-language architectures.
- These models still fall short of human experts in interpreting medical images and producing diagnostic reports, even at a preliminary level.
- Effective AI systems need access to diverse, comprehensive datasets to improve learning and generalization.
- A significant challenge is the lack of standardized evaluation metrics for AI-generated reports. Metrics that accurately reflect clinical accuracy and utility are crucial for integrating AI into clinical practice effectively.

Summary Statement While artificial intielligence vision-language models in radiology report generation have shown rapid progress, they are still unable to consistently produce high-quality reports. Key areas needing improvement include data diversity, model training, and the development of standardized evaluation metrics that reflect clinical relevance and utility.

## Introduction

Radiology reports serve as medical documentation and critical tools for diagnosis, therapeutic planning, and continuity of care. Despite advances in imaging technologies, generating precise and clinically insightful reports remains challenging and heavily dependent on radiologists’ expertise. This dependence, combined with increasing imaging volumes and radiologist shortages, creates a workflow bottleneck. In the United States, non-invasive diagnostic imaging utilization increased by 85% among Medicare enrolees and 66% among commercially insured adults between 2003 and 2016, while the number of practicing radiologists grew by only 13%, underscoring a widening workforce gap projected to persist for decades.^1^^,^^2^

Recent breakthroughs in artificial intelligence (AI), particularly foundation models like the GPT series^3^ and transformer architectures,^4^ offer potential to alleviate this gap. Unlike traditional deep learning models requiring task-specific datasets, foundation models trained on large-scale data generalize across multiple domains, enabling zero-shot and few-shot learning scenarios.^5^^,^^6^ These models leverage labelled and unlabelled data to detect patterns in medical imaging with applications in radiology report generation.

AI models capable of producing preliminary radiology reports represent a new frontier. By integrating natural language processing (NLP) and large language models (LLMs) with vision architectures, vision-language models (VLMs) can interpret images and translate findings into clinically relevant text.^7^^,^^8^ These models are designed to emulate radiologists’ analytical depth, enhancing the accuracy and relevance of generated reports by bridging image interpretation and narrative synthesis.

Yet, challenges remain. Variability in datasets, annotation uncertainty, and the lack of standardized evaluation metrics complicate consistency and comparison across models. Moreover, medical images’ complexity and radiology’s nuanced language require models to identify subtle anomalies and articulate them appropriately.

Given rapid advances, a comprehensive review is timely. This review surveys recent VLMs for radiology report generation, examining methodologies, performance, and capabilities against clinical requirements. Most datasets focus on chest radiography, with CT, MRI, and ultrasound underrepresented, limiting this review to chest X-ray-centric progress while recognizing the need for broader multimodal studies.

## Preliminaries

Radiology report generation requires integrating visual and textual modalities. VLMs address this by embedding imaging data and corresponding descriptions into a unified space.^9^^,^^10^

### Vision-language model architecture

VLMs use dual encoders, one for images and one for text, that map inputs into high-dimensional features. Pre-training aligns paired image–text examples in a shared embedding space, enabling downstream report generation without full supervision.

#### Image encoders

Early designs relied on Convolutional Neural Networks (CNNs) : VGG^11^ and EfficientNet^12^ extract hierarchical features, while ResNet^13^ introduced residuals to overcome vanishing gradients. Antialiased rect-2 blur pooling^14^ further improved shift-invariance, enhancing multimodal benchmarks.^15^^,^^16^

Recently, transformers have become the prevailing choice. ViT^17^ and Swin^18^ process image patches with self-attention, using a “class” token for global context and positional embeddings for spatial order. This captures long-range dependencies absent in CNNs and underpins state-of-the-art VLMs. Extensions include CrossViT (multi-scale),^19^ Segmenter (segmentation),^20^ detection^21^ and Transformer-in-Transformer.^22^ With scalability, generalization, and global context capture, transformers excel on large-scale datasets.^17^

#### Text encoders

Most linguistic modules adopt transformer encoder–decoder stacks. The encoder embeds tokenized report text with positional encodings, then applies multi-head scaled dot-product self-attention^4^^,^^23^ to capture syntactic and semantic dependencies. Outputs flow through feed-forward layers with residuals and normalization.

During generation, the decoder uses masked self-attention on prior tokens and encoder-decoder cross-attention to condition predictions on both text and image features, enabling coherent, clinically detailed narratives. Recent models such as Med-Flamingo^24^ follow this backbone, often initialized from LLMs like LLaMA-2,^25^ reducing domain-specific fine-tuning needs.

#### Volumetric extensions in VLMs

While most VLMs process 2D images, volumetric methods promise greater accuracy. 3D CNNs learn continuity across slices,^26^ while 3D ViTs partition volumes into cubic tokens with 3D self-attention.^27^ When full 3D use is infeasible, multi-view fusion combines features from axial, coronal, and sagittal planes.^28^ Differentiable volume rendering learns implicit radiance fields for fine-grained image-text alignment.^29^ Mesh-based surface models convert anatomy into graphs for vertex/edge-level lesion localization.^30^ Though beyond this review’s scope, these volumetric and multi-view strategies represent promising directions for future VLMs.

### Foundations of VLM pre-training

VLMs acquire cross-modal understanding by jointly exposing vision and language encoders to large collections of paired images and text. This stage is defined by 2 interdependent design choices: (1) how the modalities are architecturally integrated and (2) which self-supervised, weakly supervised, or supervised learning signals guide the joint representation.

#### Multimodal integration architectures

**Figure 1.**
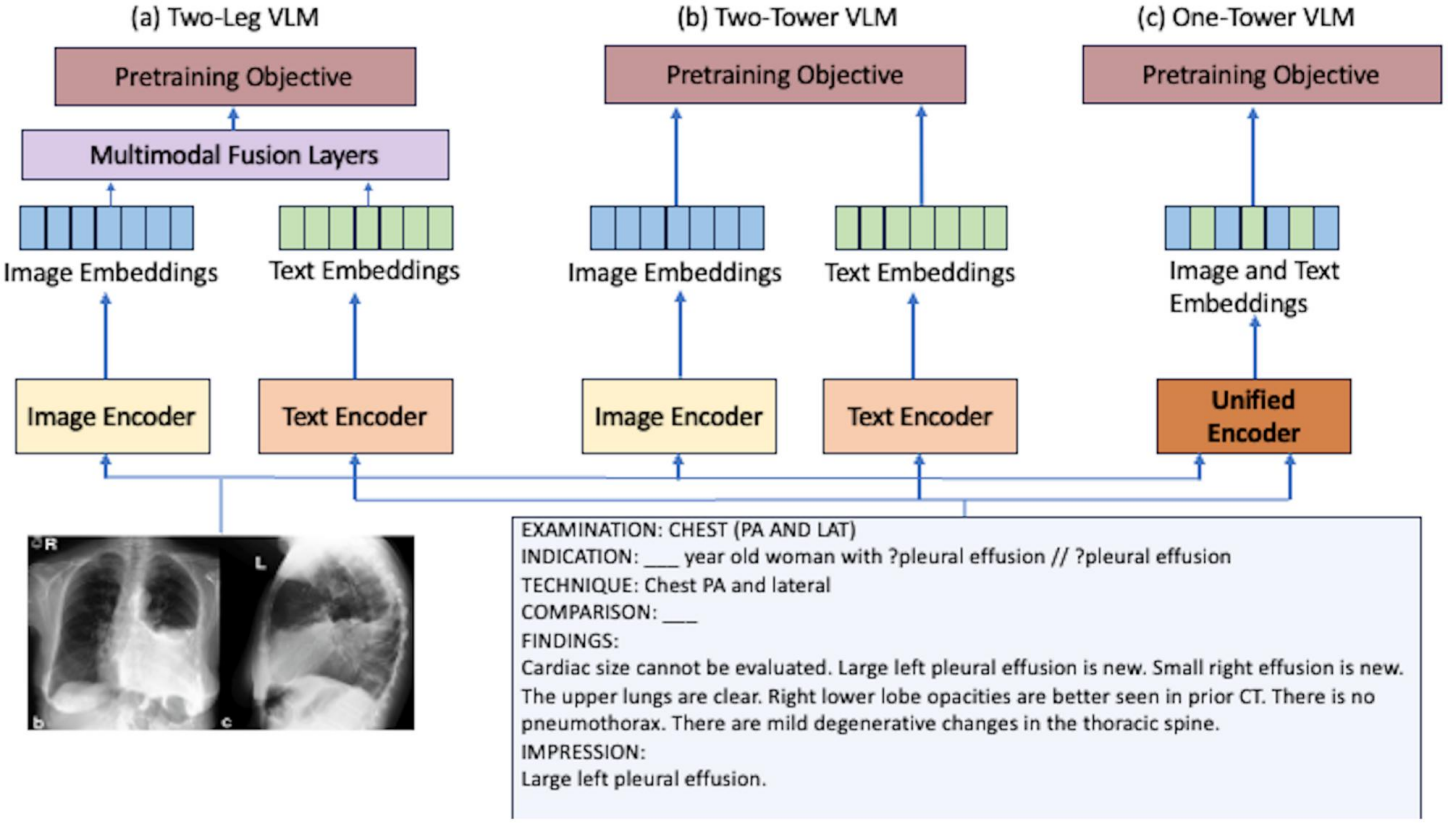
Pre-training architecture variants for vision-language models (VLM) with real-world image and text inputs^88^: (A) 2-leg VLM with interleaved multimodal fusion layers, (B) 2-tower VLM with independent image and text encoders aligned via contrastive loss, (C) 1-tower VLM with a unified encoder processing interleaved vision and language tokens.

Vision and language integration strategies can fall into 3 architectural families (Figure 1), each reflecting different theories of modality interaction:

1. Two-Tower (Dual-Encoder) Models
  Image and text encoders operate independently, producing separate embeddings only brought into alignment by a contrastive objective at the final stage. DeViSE^31^ pioneered this approach in vision–language by mapping image features into a word2vec embedding space, and CLIP^32^ demonstrated its power at scale, achieving strong zero-shot transfer by contrastively aligning 400 million image–text pairs.^33^^,^^34^
2. Two-Leg (Co-Attention) Models
  To facilitate deeper cross-modal reasoning, two-leg models intersperse co-attention or fusion layers between otherwise separate towers. ViLBERT^10^ and LXMERT^35^ extend BERT‐style text transformers with interleaved cross-attention blocks: image region features attend to text tokens and vice versa, allowing iterative refinement of joint representations before any final alignment.^24^^,^^36^
3. One-Tower (Unified) Models
  One-Tower architectures collapse vision and language inputs into a single Transformer stream. Approaches such as UNITER^37^ and Florence^38^ interleave patch and token embeddings from the first layer onward, enabling every attention head to consider both visual and textual cues simultaneously. Such pervasive fusion enriches multimodal context across layers, boosting tasks like visual question answering and report generation, at the cost of a large parameter footprint.^38^^,^^39^

#### Learning objectives for pre-training

During pre-training, VLMs optimize one or more self- or weakly supervised objectives, depending on available supervision and downstream use.

1. Contrastive Alignment: Using paired image–text data, models like CLIP^34^ employ an InfoNCE loss^33^ to pull matched pairs together and push apart mismatches. This requires minimal supervision, scales to large datasets, and yields global semantic representations for zero-shot transfer.
2. Masked Generative Modelling: Input patches or text tokens are masked and reconstructed from context.^40–42^ Encoders produce latent features that decoders reconstruct with mean squared error (visual) or cross-entropy (textual) losses, encouraging models to infer texture, form, and linguistic relations. This bidirectional reasoning fosters context-aware multimodal representations without external labels.
3. Region-Level Grounding: With localized annotations (eg, bounding boxes or phrase grounding), models align visual subregions with text spans.^43^^,^^44^ This fine-grained supervision improves interpretability and supports tasks requiring precise localization.

State-of-the-art VLMs often combine these objectives to balance global alignment, detailed reconstruction, and precise grounding, achieving robust generalization and interpretability for applications such as radiology report generation.

### Task-adaptive fine-tuning

**Figure 2.**
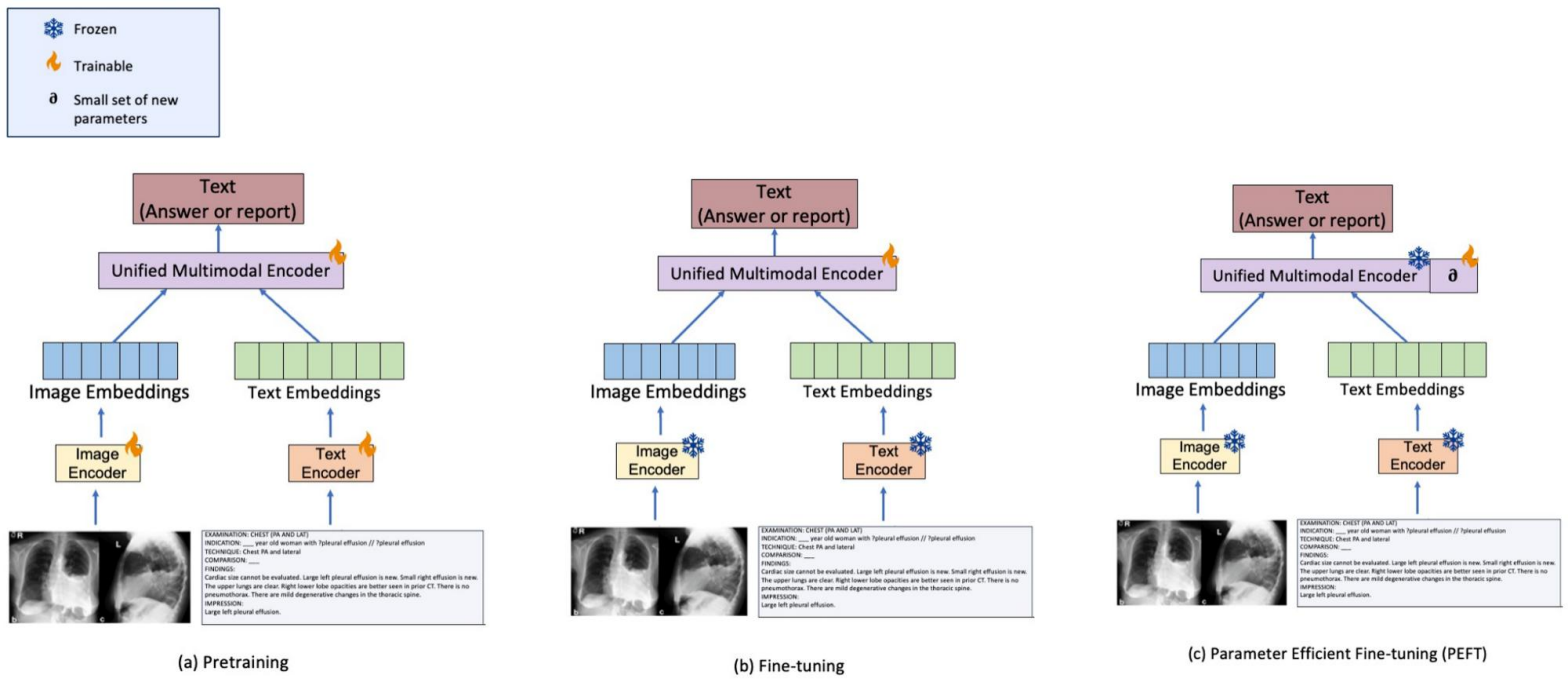
Framework comparison for vision-language models with real-world image and text inputs^88^: (A) pre-training, (B) fine-tuning, and (C) parameter-efficient fine-tuning. Red box presents the frozen parameters.

After pre-training, VLMs require adaptation for tasks like report generation or pathology classification. Two strategies dominate: (1) full-parameter tuning, updating all model weights and (2) parameter-efficient tuning, restricting updates to small auxiliary components. Figure 2 illustrates (A) pre-training, (B) full fine-tuning, and (C) parameter-efficient fine-tuning.

#### Full-parameter tuning with task heads

In this regime, both vision and language encoders are retrained end-to-end on labelled radiology data. Task heads map embeddings to outputs like report sequences or diagnostic labels. Localization tasks employ Region of Interest pooling,^45^ while cross-modal attention modules^46^ can enhance alignment for caption correction or question answering. Robustness may be improved with adversarial training^47^ to counter noise and distribution shifts. Although highly accurate with sufficient data, this approach is computationally expensive and risks catastrophic forgetting.

#### Parameter-efficient tuning

To reduce cost, parameter-efficient methods update only a fraction of weights. Adapters^48^ insert trainable bottlenecks between Transformer layers, while BitFit^49^ adjusts only bias terms, often recovering most performance. LoRA^50^ applies low-rank updates, approximating key weight directions with minimal parameters. These techniques allow efficient adaptation to radiology tasks on limited hardware and data while retaining strong performance.

### VLM evaluation protocols

Two main protocols evaluate a VLM’s generalization without extensive retraining. Zero-shot evaluation tests tasks with no fine-tuning data and reflects the model’s ability to perform tasks it has never been trained on, relying on broad pre-training to match images to report phrases or generate unseen pathology descriptions. Metrics include retrieval accuracy, natural language generation (NLG) scores such as BLEU^51^ and CIDEr, and clinical factuality measures like RadGraph F1^36^ that assess the correctness of extracted entities.

**Figure 3.**
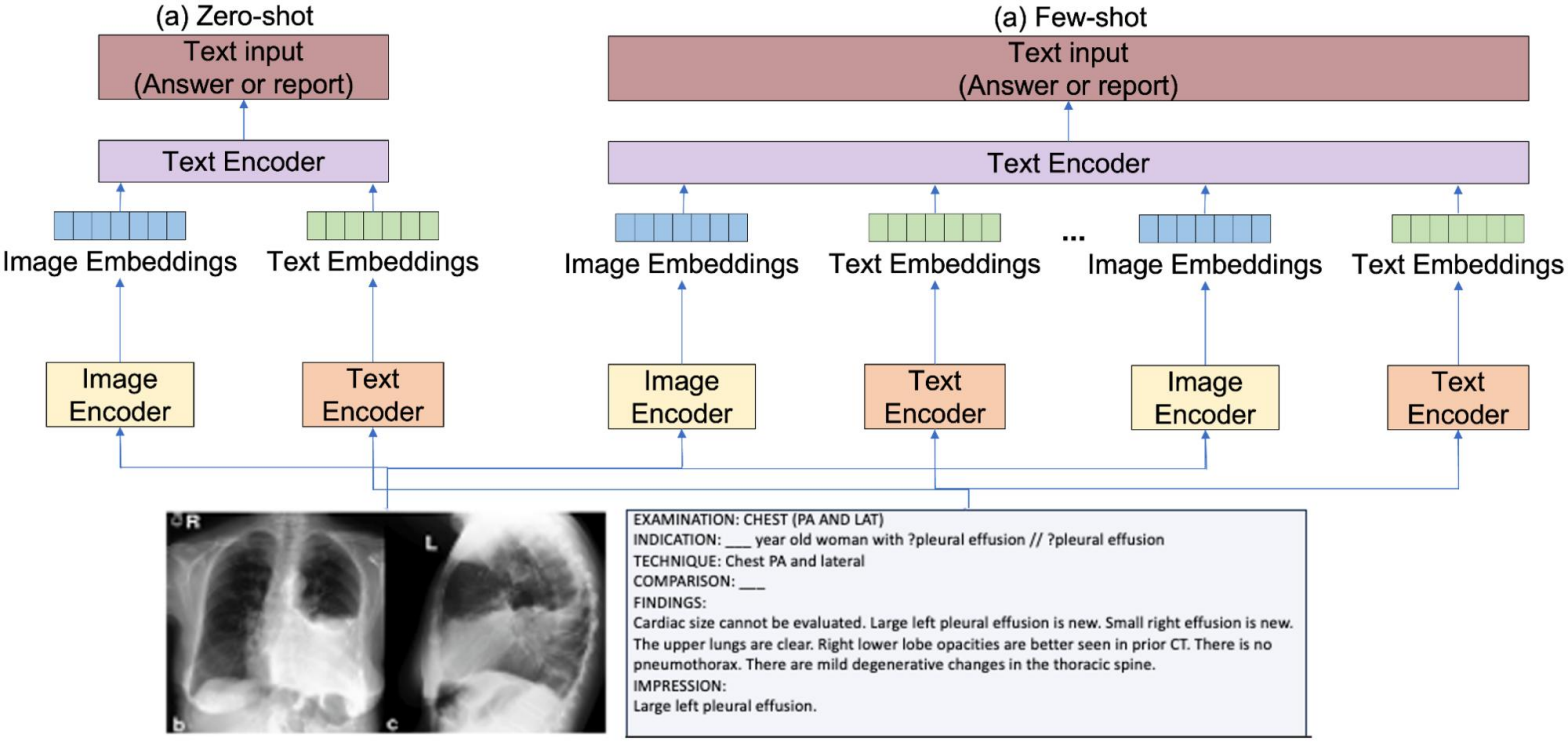
Description of the framework for zero-shot prediction and few-shot prediction with real-world image and text inputs.^88^

Few-shot evaluation uses 10-100 annotated cases and assesses the model’s ability to adapt with minimal labelled examples through lightweight modules (adapters, bias terms) or task-specific heads. This balances limited annotation with performance gains over zero-shot while avoiding full supervision. In medical imaging, where labels are scarce, strong few-shot results underscore VLMs’ utility for rapid deployment across diverse clinical tasks. Figure 3 illustrates the distinction between these frameworks.

**Figure 4.**
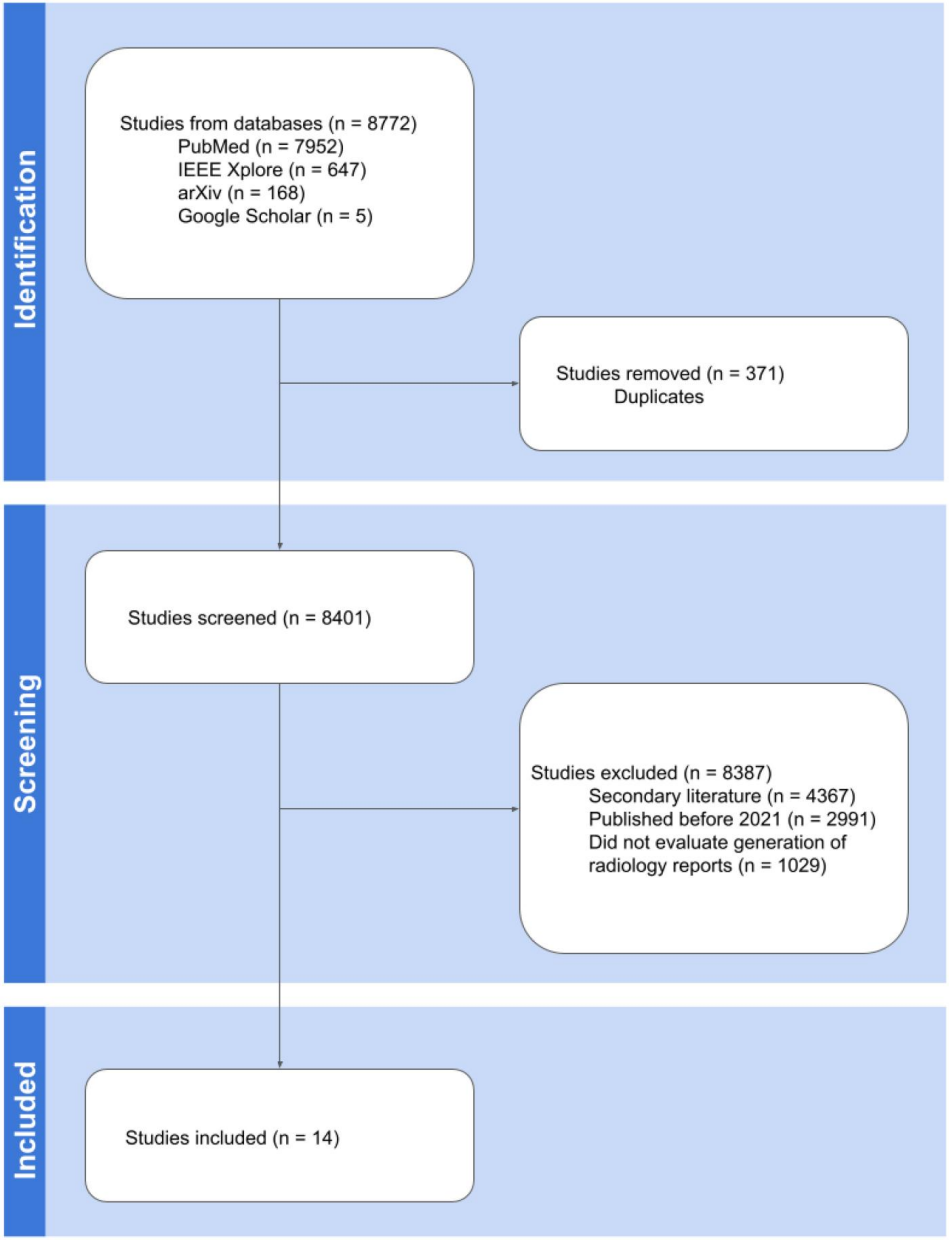
Flowchart of methods.

### Comparative analysis of VLM design choices

The design of VLMs, including architecture, pre-training objectives, fine-tuning strategies, and evaluation modes, shapes radiology report generation effectiveness.

#### Architecture paradigms

Dual-/two-tower: CXR-RePaiR^34^ applied a CLIP-style encoder to MIMIC-CXR, achieving CheXbert^52^ macro-F1 = 0.540 (±0.227) and reducing generation time by ∼70% compared to autoregressive models, though BLEU-4 was low (∼0.1). ALIGN^39^ scaled this paradigm to 1.8B image–text pairs, demonstrating adaptability for retrieval/report tasks.

Unified one-tower: UNITER^37^ fused image and text embeddings for stronger alignment. Florence^38^ extended this to foundation scale, and in CXR adaptation, BioViL-T^53^ improved CheXbert F1 (0.317) and RadGraph F1 (0.495) over retrieval-only systems.

#### Pre-training objectives

Contrastive alignment: CLIP-style InfoNCE^33^ enables label-efficient transfer; CXR-RePaiR^34^ reached macro-F1 ≈ 0.54, though rare findings remain difficult to capture.^54^

Masked modelling: Reconstruction objectives, inspired by SimMIM^40^ and MAE,^55^ boost semantic coverage. METransformer^56^ achieved METEOR = 0.152, CIDEr-D = 0.362, while M2 Transformer^57^ with factuality penalties reached CIDEr-D = 0.492-0.509.

Region–word alignment: UNITER^37^ formalized region–phrase matching; applied to CXR, BioViL-T^53^ achieved RadGraph F1 = 0.495.

Meta-masking: Modality-agnostic objectives^58^ remain unexplored in radiology but may aid heterogeneous datasets.

#### Fine-tuning strategies

Full fine-tuning: Updating all parameters on MIMIC-CXR (∼300k pairs)^59^ yields peak metrics but is compute-intensive.

Parameter-efficient tuning: Adapters^48^ recover >95% of full performance with ∼1000 labelled CXRs (eg, MAIRA-1^60^). BitFit^49^ updates <0.1% of weights with competitive BLEU/ROUGE, and LoRA^50^ in Med-PaLM-M^8^ retained >90% of performance using 10% of data.

#### Evaluation modes

Zero-shot: CXR-RePaiR^34^ reached CheXbert macro-F1 ≈ 0.54 without labelled data, but BLEU-4 remained low (∼0.1).

Few-shot: BioViL-T^53^ improved RSNA pneumonia classification from F1 = 0.706 (zero-shot) to 0.730 with 1% labelled data.

Fully supervised: Masked models dominate lexical scores, eg, METransformer^56^ (METEOR = 0.152, CIDEr-D = 0.362), R2GenGPT^61^ (BLEU-4 = 0.134), and M2 Transformer fCEN^57^ (CIDEr-D = 0.509), illustrating gains from full supervision at higher annotation cost.

## Methods

### Search strategy

A focused literature search was conducted in 2024 for studies published from 2020 onward to capture recent advances in AI models for radiology report generation. Databases searched included PubMed, IEEE Xplore, arXiv, and Google Scholar using the terms: “AI radiology report generation,” “deep learning radiology reports,” “radiology foundation model,” and “radiology vision-language model.” Models referenced in selected papers were also included. This yielded 8772 studies, reduced to 8401 after removing duplicates (Figure 4).

### Inclusion criteria

Studies were eligible if published in or after 2021, explicitly evaluated radiology report generation capabilities, and employed recognized evaluation metrics. Priority was given to those using RadGraph F1^36^ and RadCliQ,^62^ though traditional NLG metrics (eg, BLEU^51^) and custom criteria were also accepted.

### Exclusion criteria

Excluded were reviews, editorials, non-original research, studies unrelated to report generation, and those published before 2021. After applying these criteria, 14 studies remained (Table 1).

**Table 1.** Summary of 14 included studies.

Author (year)	Model name	Data used for training	Architecture	Computing resources	NLG metrics	Labeller	CE metrics (as reported)	Human evaluation	Training process	Performance summary
Endo et al. (2021)^34^	CXR-RePaiR	MIMIC-CXR (reports and CXRs)	CLIP-based dual encoder (ViT-B/32 image encoder + 12-layer standard transformer text encoder [Two-Tower (contrastive VLM)]	NVIDIA Titan X	BLEU-2; semantic similarity (BERTScore)	CheXbert	F1-14: 0.352 (CheXpert external), 0.274 (MIMIC-CXR)	No	Contrastive pretraining (natural images), then radiology pairs; retrieval of top-k sentences (“Select”)	Retrieval approach achieves higher clinical F1 than generative baselines and is robust out-of-distribution (CheXpert).
Miura et al. (2021)^57^	M2 Transformer	MIMIC-CXR subset (≈152k pairs) + Openi CXR data	DenseNet-121 image encoder + Transformer text decoder (reinforcement learning with factual completeness (factENT) and consistency (factENTNLI) rewards). [Two-Leg (encoder → decoder, generative)]	NVIDIA Titan XP	BLEU-4; CIDEr-D	CheXbert	Micro F1-14: 0.567	No	Supervised pretrain → RL with BERTScore + NLI-style rewards	Strong CIDEr-D; RL markedly improves CheXbert F1 and factuality vs. baselines.
Chen et al. (2020)^64^	R2Gen (Memory-driven Transformer)	MIMIC-CXR; IU-Xray	Transformer with Relational Memory for text decoder; ResNet-101 image encoder [Two-Leg]	Not reported	BLEU-1/2/3/4; METEOR; ROUGE-L	CheXpert	F1-14 (CheXpert)	No	Adam optimizer; memory-augmented decoding; conditional layer norm with memory	Improves NLG metrics; memory helps long-range coherence in reports.
Wang et al. (2023)^72^	ChatCAD	300 chest X-rays + prompts	LLM-assisted CAD pipeline (visual CAD + GPT-3/ChatGPT prompting) [One-Tower (LLM-centric unified interface)]	Not reported	(NLG not primary)	CheXpert	Diagnostic F1 ↑ ∼16.4 points	No	LLM-CAD integration; interactive prompting	Improves CAD diagnostic accuracy; used CheXpert for clinical labeling.
Bannur et al. (2023)^53^	BioViL-T	MIMIC-CXR v2 for pretraining	CNN image encoder + Transformer fusion (with temporal/spatial encodings) + CXR-BERT text encoder. [Two-Tower (contrastive + masked LM)]	8 NVIDIA Tesla V100s (32 GB each)	BLEU-4; ROUGE	CheXbert	CheXbert finding-level metrics	No	Contrastive learning + masked-LM variants	Strong factuality trends via CheXbert; alignment improves localization sensitivity.
Wang et al. (2023)^56^	METransformer	MIMIC-CXR; IU-Xray	ViT image encoder + expert bilinear Transformer encoder-decoder (learnable “expert” tokens) [Two-Leg]	2 NVIDIA GeForce RTX 3090	BLEU-1/2/3/4; METEOR; ROUGE-L; CIDEr	CheXpert	F1-14 (MIMIC-CXR, CheXpert)	No	Expert tokens; orthogonality loss; metrics-based expert voting	Top METEOR (0.152) and strong CIDEr-D; consistent gains on MIMIC and IU-Xray.
Tanida et al. (2023)^69^	RGRG (Region-Guided)	MIMIC-CXR (subset)	Faster R-CNN + ResNet-50 (image encoder + detector); GPT-2 Medium text decoder (region-conditioned) [Two-Leg (region-conditioned)]	NVIDIA A40	BLEU-1/2/3/4; METEOR; ROUGE-L; CIDEr-D	CheXbert	CheXbert F1-14	No	Three-stage training: detect regions → select salient regions → generate region sentences	Competitive NLG; better RadGraph-style coherence than global captioners.
Hyland et al. (2024)^60^	MAIRA-1	MIMIC-CXR (+institutional text for augmentation)	RAD-DINO CXR encoder + Vicuna-7B LLM via learnable adapter (MLP); instruction-tuned [One-Tower (LLM backbone with visual adapter)]	Not reported	ROUGE-L; BLEU-4; METEOR	CheXbert	F1-5; F1-14 (CheXbert); RadGraph F1; RadCliQ	Yes	Standard autoregressive cross-entropy; instruction tuning; augmentation	Improves RadCliQ and lexical metrics; manual review shows higher fluency and accuracy.
Nicolson et al. (2023)^65^	CvT-21 DistilGPT2	272 859 CXRs (MIMIC-CXR-JPG)	CvT-21 encoder image encoder→ DistilGPT-2 text decoder with cross attention (warm-started) [Two-Leg]	4× NVIDIA P100 (16 GB each)	BLEU-1/2/3/4; METEOR; ROUGE-L; CIDEr	CheXbert	CE F1 + 8.3%; F1-14 (CheXbert)	No	Teacher forcing; CE; AdamW; warm-start both encoder/decoder	Lightweight hybrid with solid gains in CE and NLG.
Tu et al. (2023)^8^	Med-PaLM-M	MIMIC-CXR subset (+other medical corpora)	Vision Transformer image encoder + PaLM-based LLM (84B) with LoRA adapters; instruction-tuned on CXR/CT + medical text. [One-Tower]	Not reported	BLEU-1; BLEU-4; METEOR; ROUGE-L	CheXbert	F1-5; F1-14 (micro/macro)	Yes	Fine-tuning (LR 5e-5); multi-size variants	40.5% of model reports preferred over originals; strong CheXbert F1.
Yan et al. (2023)^73^	WCT (Style-aware/Weighted Clinical Translation)	MIMIC-CXR + RadGraph	DenseNet-121 image encoder + Transformer decoder; style prompting with GPT-3.5-turbo [Two-Leg with external prompting]	AWS g5.2× large instance with one NVIDIA A10G Tensor Core	BLEU-2; BERTScore	CheXbert	F1-14 (CheXbert); RadGraph F1	No	Structured representation + prompting; style control	Reduces omissions; human raters struggled to distinguish AI vs human reports.
Wu et al. (2023)^66^	RadFM	MedMD (16M pairs) → RadMD (3M radiology pairs)	3D ViT (12-layer, 768-dim) + Perceiver Transformer (6-layer, 32 × 5120 latent) + MedLLAMA-13B LLM as text decoder. [One-Tower (unified multimodal backbone)]	32× NVIDIA A100	BLEU-1; ROUGE-1; BERT-Sim; UMLS precision/recall	None	No CheXpert/CheXbert metrics; human eval only	Yes	Pretrain on MedMD → finetune on RadMD; evaluate on RadBench	Strong across multimodal tasks; handles 2D/3D; occasional localization misses.
Tanno et al. (2023)^71^	Flamingo-CXR	472 824 CXRs (multi-site)	Flamingo-style VLM, adapted for CXR (NFNet-F6 vision encoder + Perceiver Resampler + Chinchilla LLM with gated cross-attention layers) [One-Tower (Flamingo-style cross-attention)]	TPUv4 instances with large-scale parallelism (1536 chips, 15 days) using JAX/Haiku, ZeRO sharding, and mixed-precision training (bfloat16).	BLEU-4; ROUGE-L; CIDEr	CheXpert	F1-14 (CheXpert); RadGraph F1	Yes	Regularization and adaptation for radiology; expert study	On par/better than humans in 43% (United States) and 60% (India); improves further with clinician edits.

### Data extraction and synthesis

From each study, data were extracted on model name, report generation capabilities, training datasets, evaluation metrics, and performance outcomes. These were synthesized to provide a comparative overview of model effectiveness, dataset usage, and evaluation strategies, enabling discussion of recent trends in AI-driven radiology report generation.

## Discussion

### Training datasets

AI radiology report generation effectiveness depends on datasets with diverse pathologies and demographics. Standardized train: test splits have been attempted, though subsets vary. Of 14 surveyed models, 11 were trained exclusively on chest radiographs (mainly MIMIC-CXR). Miura et al.’s M2 Transformer, trained on 152 173 MIMIC-CXR images, improved factual completeness and consistency,^57^^,^^63^ while Endo et al’s CXR-RePaiR, trained on 300 000 MIMIC-CXR pairs, refined accuracy with CLIP.^34^ Several studies supplemented MIMIC0CXR with additional chest radiograph datasets. Miura et al. and Chen et al. added Open I^64^ and IU X-Ray^57^ chest radiographs to increase sample size and stylistic diversity of reports. Nicolson et al. used MIMIC-CXR-JPG, a JPEG-converted variant of MIMIC-CXR, while methods emphasizing structured supervision, like Weighted Clinical Translation (WCT) and **R**egion-**G**uided Radiology **R**eport **G**eneration (RGRG), relied on RadGraph-annotated subsets of MIMIC-CXR.^65^ Large-scale foundation models broadened beyond CXR alone: Med-PaLM-M^8^ incorporated multimodal biomedical corpora, MAIRA-1^60^ added institutional radiology reports, and RadFM^66^ trained on MedMD and RadMD, web-scale multimodal collections spanning 2D and 3D medical imaging. These datasets enrich pathology and domain coverage, but they are either limited in scale and standardization, modality-specific, or proprietary, limiting their role as shared benchmarks compared with MIMIC-CXR. Broader modality coverage has been explored in systems such as Med-PaLM M, which extends beyond chest radiographs to include additional medical imaging domains^8^ and BioViL-T (POCUS ultrasound),^53^ though no systems yet leverage MRI (such as BraTS^67^ or other cardiac MRI data) or lesion-annotated CT (LIDC-IDRI^68^).

Different models highlight complementary strengths. R2GenGPT^61^ (ViT + GPT-2 with RL) led BLEU/ROUGE-L; METransformer^56^ (masked patches/tokens) excelled on METEOR/CIDEr-D; M2 Transformer fCEN^57^ maximized CIDEr-D. CXR-RePaiR-2 and Select^34^ combined lung segmentation with CLIP, cutting hallucinations by 15%. BioViL-T^53^ tied Faster R-CNN to report phrases (RadGraph F1: 0.495). R2GenCMN^64^ used cross-modal memory for coherence; CvT-21DistilGPT2^65^ showed small hybrids can match larger systems. Med-PaLM-M^8^ fine-tuned an 84B LLM with LoRA adapters, recovering >90% of full performance using 10% of data.

Several works emphasize trust and interactivity. Tanida et al.’s human-in-the-loop design improved transparency,^69^^,^^70^ RadFM^66^ integrated radiologist-rated scores for factual accuracy, while Flamingo-CXR^71^ and ChatCAD^72^ generalized few-shot to new pathologies. WCT^73^ embedded domain priors, reducing omissions by 20%. MAIRA-1^60^ balanced strong language scores (BLEU-1: 0.392, ROUGE-L: 0.333) with clinical accuracy (CheXbert F1-5: 0.557, F1-14: 0.386).

Despite progress, performance lags for rare pathologies, reflecting reliance on chest radiographs (eg, MIMIC-CXR).^63^^,^^74^ Expanding to CT, MRI, and volumetric data with standardized splits will be critical.

### Evaluation metrics

#### Natural language generation (NLG) metrics

NLG metrics evaluate similarity between generated reports and references through word overlap, structural alignment, or semantic matching. The most widely used are Bi-Lingual Evaluation Understudy (BLEU), Recall-Oriented Understudy for Gisting Evaluation (ROUGE), Metric for Evaluation of Translation with Explicit Ordering (METEOR), Consensus-based Image Description Evaluation (CIDEr), and Bidirectional Encoder Representations from Transformers Score (BERTScore).

BLEU measures average n-gram overlap, with BLEU-1 to BLEU-4 extending from unigrams to four-grams, and applies a brevity penalty to discourage short outputs.^51^ ROUGE emphasizes recall: ROUGE-N counts n-gram matches, while ROUGE-L uses the longest common subsequence to balance recall and precision.^75^ METEOR incorporates stemming, synonym matching, and word-order penalties, computing a weighted harmonic mean that often aligns better with human judgements than BLEU.^76^ CIDEr scores consensus against multiple references using TF-IDF-weighted n-grams (1-4), with CIDEr-D correcting for repetition and length exploitation.^77^ BERTScore instead uses contextual embeddings from BERT to calculate cosine similarity, directly capturing semantic alignment and correlating strongly with human evaluations.^52^^,^^78^

Despite their prevalence, inconsistent implementations (eg, BLEU n-gram size) hinder cross-study comparability, and these metrics emphasize linguistic fidelity rather than clinical accuracy. As Babar et al. noted, standardized templates can inflate scores without ensuring diagnostic quality,^79^ motivating the use of clinical efficacy (CE) metrics.^70^

On the MIMIC-CXR dataset, BLEU (especially BLEU-4) and ROUGE remain most used (Table 2). R2GenGPT achieved top BLEU and ROUGE scores, while METransformer excelled in METEOR and CIDEr. MAIRA-1 performed competitively, ranking near R2GenGPT and CvT-21DistilGPT2 in BLEU-1 and maintaining high ROUGE-L. Architectures emphasizing contrastive alignment (eg, CXR-RePaiR-Select) showed strong zero-shot retrieval but weaker generative fidelity, whereas reconstructive masked-modelling designs (eg, METransformer, R2GenGPT) achieved higher METEOR and CIDEr-D scores at the cost of occasional hallucinations. RadGraph F1, reported only for models using the RadGraph framework (BioViL-T, RGRG, R2GenGPT), offers a complementary measure of clinical correctness.

**Table 2.** Performance of vision‐language models on the MIMIC-CXR test set across natural language generation metrics.

	BLEU				METEOR	ROUGE-L/ROUGE*	CIDEr/CIDER-D*
**Model name**	**BLEU-1**	**BLEU-2**	**BLEU-3**	**BLEU-4**			
CXR-RePaiR-2^34^		0.256		0.021			
CXR-RePaiR-Select^34^		0.274					
M2 Transformer fCE^57^				0.111			0.492*
M2 Transformer fCEN^57^				0.114			0.509*
R2GenCMN^64^	0.353	0.218	0.145	0.103	0.142	0.277	
BioVilT^53^		0.2131		0.092		0.296*	
METransformer^56^	0.386	0.25	0.169	0.124	0.152	0.291	0.362
RGRG^69^	0.373	0.249	0.175	0.126	0.168	0.264	0.495*
CvT-212DistilGPT2^65^	0.3918	0.2454	0.1685	0.1236	0.1525	0.2846	0.3614
Med-PaLM M^8^		0.3231		0.115		0.2749	0.2617*
RadFM^66^	0.1943					0.2618*	
Flamingo-CXR^71^				0.101		0.297*	0.138
ChatCAD^72^							
WCT^70^		0.18					
R2GenGPT^61^	0.411	0.267	0.186	0.134	0.16	0.297*	0.269*
MAIRA-1^60^	0.392			0.142	0.333	0.289	

Higher values indicate stronger word‐ and phrase‐level fidelity and semantic reconstruction. In columns reporting ROUGE-L/ROUGE and CIDEr/CIDER-D, values marked with an asterisk denote ROUGE and CIDER-D, respectively.

Future evaluations should therefore combine surface-level metrics (BLEU, ROUGE, METEOR, CIDEr-D, BERTScore) with structured clinical metrics (RadGraph F1) to comprehensively benchmark radiology report generation.

#### Clinical efficiency (CE) metrics

To assess clinical accuracy, many studies use the CheXpert and CheXbert labellers, which detect 14 clinical observations in radiology reports and compute precision, recall, and F1 scores. Performance is typically reported as F1-14 (all 14 observations) or F1-5 (five key findings: atelectasis, cardiomegaly, consolidation, oedema, pleural effusion). CheXbert, enhanced with a biomedical BERT model, improves accuracy over CheXpert, leading researchers like Miura et al. to prefer it.^57^

Beyond labellers, the RadGraph Benchmark employs the DYGIE++ information extraction framework with PubMedBERT pre-training to capture detailed entities (diagnoses, anatomical parts) and their relations. Clinical accuracy is measured using the RadGraph F1 score, which quantifies overlap of entities and relationships between generated and reference reports.^80^

The Radiology Report Clinical Quality (RadCliQ) metric, developed later by the RadGraph team, combines BLEU, BERTScore, CheXbert vector similarity (S_meb), and RadGraph F1. Studies comparing metrics against radiologist evaluations found RadCliQ most closely aligned with human judgement, with RadGraph F1 as the strongest individual correlate.^79^

Because NLG metrics often fail to capture clinical correctness, most studies incorporate CE metrics, usually CheXpert or CheXbert scores. However, methodologies vary (eg, labeller choice, whether F1-14 or F1-5 is used, and whether scores are micro- or macro-averaged). A slight majority of studies favour CheXbert, with F1-14 the most common metric. Standout F1-14 scores were reported by models such as Flamingo-CXR, Med-PaLM-M, and RGRG (Table 3).

**Table 3.** Clinical efficiency of vision-language models on the MIMIC-CXR test set under CheXpert and CheXbert labellers.

	MIMIC-CXR test set with CheXpert labeller	
Model	F1-5	F1-14
Flamingo-CXR^71^	0.58	0.519
ChatCAD (GPT-3)^72^	0.591	
ChatCAD (ChatGPT)^72^	0.605	
METransformer^56^		0.311
R2GenGPT^61^		0.389

	MIMIC-CXR test set with CheXbert labeller	
Model	F1-5	F1-14
M2 Transformer fCE (original paper)^57^	0.567	
M2 Transformer fCEN (original paper)^57^	0.567	
CXR-RePaiR-2^34^		0.256
CXR-RePaiR-Select^34^		0.274
BioViLT^53^		0.317
R2Gen CMN^64^		0.278
RGRG^69^	0.547	0.447
CvT-212DistilGPT2—micro^65^		0.442
CvT-212DistilGPT2—macro^65^		0.307
Med-PaLM-M—micro^8^	0.5788	0.5356
Med-PaLM-M—macro^8^	0.5160	0.3993
MAIRA-1^60^	0.557	0.386

F1-5 (5 key findings) and F1-14 (all 14 observations) are shown for each model.

While RadGraph F1 offers broader clinical coverage beyond the 14 observations, its adoption has been limited. Similarly, despite its potential as a comprehensive benchmark, RadCliQ has only been applied by its developers in a single study so far.^62^

Surveyed models can be grouped by optimization strategy: masked modelling, contrastive alignment, architectural scaling, and domain- or quality-specific objectives. Each reflects a trade-off between linguistic fidelity and clinical correctness.

Masked modelling: METransformer^56^ achieved the highest METEOR (0.152) and strong CIDEr-D (0.362), while R2GenGPT secured top BLEU-4 (0.134) and higher BERTScore with explicit cross-attention. The M2 Transformer fCE/fCEN variants^57^ reached the best CIDEr-D (0.492/0.509) while maintaining factual completeness, and R2GenCMN^64^ improved coherence via cross-modal memory. Overall, masked-modelling excels in METEOR and CIDEr-D but lags in RadGraph F1.

Contrastive alignment: CXR-RePaiR-2/Select^34^ delivered moderate BLEU-4 (∼0.10) but strong zero-shot retrieval and robustness. BioViL-T,^53^ with region-word alignment, achieved midrange BLEU/METEOR yet outperformed in RadGraph F1, underscoring the benefit of local alignment. These models favour entity-relation accuracy but underperform in n-gram precision.

Backbone and scaling: CvT-21DistilGPT2^65^ (∼200M parameters) maintained BLEU-2/3 ≈ 0.25, showing compact hybrids can rival larger systems. Med-PaLM-M^8^ (84B) offered only modest BLEU/METEOR gains, suggesting diminishing returns from scaling alone. In contrast, MAIRA-1^60^ balanced strong NLG (BLEU-1: 0.392, ROUGE-L: 0.333) with competitive CE scores (CheXbert F1-5: 0.557, F1-14: 0.386), demonstrating domain adaptation’s advantage over raw scale.

Specialized optimization: Flamingo-CXR^71^ and ChatCAD^72^ used few-shot prompting to produce coherent reports with limited fine-tuning. WCT^73^ embedded domain priors, reducing omission errors by 20%, while RadFM^66^ incorporated radiologist-rated quality scores, improving expert evaluations despite lower BLEU/ROUGE. These highlight that optimizing for expert judgement can diverge from optimizing surface similarity.

Metric relevance: BLEU-4 correlates poorly with radiologist ratings (*r* < 0.35), whereas RadGraph F1 correlates strongly (*r* ≈ 0.72). Models like BioViL-T^53^ and RGRG,^69^ optimized for graph alignment, rank higher in clinical accuracy, while balanced systems such as MAIRA-1^60^ show linguistic fidelity can coexist with diagnostic correctness.

No strategy dominates: masked modelling enhances semantic completeness, contrastive alignment strengthens entity-relation accuracy, scaling alone is insufficient, and domain priors improve clinical trustworthiness. The most consistent performance may emerge from hybrid approaches combining reconstruction, alignment, adaptation, and clinically informed objectives.

#### Human evaluation

Since clinician-based assessments are resource-intensive, only 4 of the 14 surveyed models employed human evaluation. Typically, 2-4 radiologists reviewed reports in blinded setups, assigning scores, ranking preferences, or noting omissions and errors. Wu et al. assessed RadFM with 3 radiologists (≥1-year clinical experience), who rated 400 test cases on a 0-5 scale (garbled to correct), averaging results.^66^ Tu et al. presented 3 Med-PaLM-M variants (12B, 84B, 562B) plus ground truth for 246 cases, with each set ranked from 1 to 4.^8^ Tanno et al. evaluated 500 cases using blinded pairwise preferences with 2 radiologists, inter-rater analysis, and an “equivalence” option, reporting both overall and inter-rater preferences.^71^

Unlike Wu et al,^66^ Tu et al,^8^ and Tanno et al^71^ performed error/omission analyses. Four radiologists independently categorized errors, noted significance, and suggested corrections, benchmarking omission and error rates against MIMIC-CXR. Tu et al. found omission rates of 0.12-0.13/report and clinical error rates of 0.25-0.29/report, comparable to prior MIMIC-CXR error rates,^81^ though not assessed by the same radiologists.^8^ Tanno et al. showed clinically significant error rates of 0.29 per AI report versus 0.10 in originals,^71^ underscoring weaknesses in complex cases.

Tu et al. reported the Med-PaLM-M (84B) variant was preferred over originals in 40.5% of cases,^8^ while Tanno et al^71^ found AI-generated reports were rated better or equal in >60%, though inter-rater agreement was low (11%-12%) in abnormal cases. Wu et al. noted RadFM was rated more favourably than peers but averaged only 1.88, between “inaccurate” and “partially informative.”^66^

Overall, human evaluation offers critical insights into preference and error patterns but remains costly, subjective, and variable. Despite some promising results, current AI models fall short of expert-level reporting, particularly for complex or uncommon findings.

## Challenges and future directions

Despite substantial progress, existing VLMs cannot be deployed autonomously for radiology report generation. Clinical adoption will require advances in data diversity, evaluation standards, explainability, and integration of human-centred design.

### Data diversity, generalizability, and training constraints

Most reviewed systems are trained on MIMIC-CXR subsets, which limits exposure to diverse scanners, institutions, demographics, and pathologies. Broader generalization will require multimodal datasets, multi-institutional curation, and improved representation across global healthcare settings. Prior work highlights significant variation in cardiovascular imaging between Low and Middle Income Countries (LMIC) and high-resource environments, which complicates AI implementation.^82^ High computational cost and domain shift remain barriers, despite progress in parameter-efficient tuning method.^48–50^ Comprehensive and heterogeneous datasets therefore remain essential for robust model development.^79^

### Evaluation standards, clinical accuracy, and explainability

The absence of standardized evaluation protocols limits comparability. Traditional NLG metrics do not capture diagnostic accuracy, prompting greater reliance on clinical efficiency measures such as CheXpert and CheXbert F1, RadGraph F1, and RadCliQ. Even these do not fully address clinical explainability. Radiologists often cannot determine which image regions support generated statements, and attention-based explanations remain inconsistent. Broader evidence from cardiovascular imaging underscores how a lack of transparency can undermine trust and patient safety.^83^ Techniques addressing spurious correlations and latent bias, like those used in GRASP-PsONet and analyses of demographic encoding in CXR classifiers,^84^^,^^85^ may improve reliability, although clinically validated solutions are limited.

### Temporal, contextual, and human-centric integration

Clinical reporting depends on comparisons with prior studies and integration of relevant Electronic Health Records (EHR) data, yet current models treat images as static, isolated inputs. Temporal alignment, progression assessment, and contextual reasoning remain largely unexplored. Ethical concerns regarding data privacy, medico-legal responsibility, and user oversight further complicate deployment.^86^ Human factors are critical since AI-generated text can increase cognitive load or introduce automation bias. Prospective reader studies, human-in-the-loop workflows, and interface designs that support radiologist decision-making will be necessary for safe adoption. Early work in physiological signal modelling, like magnetocardiographic source localization,^87^ illustrates the value of incorporating temporal and contextual information that future VLMs may emulate.

## Limitations

Current VLMs for radiology report generation are constrained by limited data diversity, inconsistent evaluation, and insufficient clinical reliability. Heavy reliance on MIMIC-CXR reduces generalizability to other modalities and underrepresented pathologies. Existing evaluation practices remain fragmented. NLG metrics emphasize linguistic overlap rather than diagnostic content, while clinical metrics such as CheXpert and CheXbert F1, RadGraph F1, and RadCliQ are unevenly applied and difficult to compare. Human evaluation remains infrequent and often reveals substantial variability in rater judgement along with persistent model errors, especially in complex imaging. These limitations make fully autonomous report generation unsafe. Persistent omission errors, hallucinations, and factual inconsistencies underscore the need for human oversight. Future work must expand multimodal datasets, standardize evaluation protocols, address explainability, and conduct multicentre validation to ensure clinically safe integration.

## Conclusion

VLMs show promise for radiology report generation but remain limited by narrow datasets, inconsistent evaluation, and variable clinical reliability. Progress in architectures and tuning is notable, yet safe adoption demands standardized metrics, broader multimodal validation, and above all, *human-in-the-loop oversight*. Properly integrated, VLMs can serve as assistive tools that enhance radiologists' efficiency and patient care.
